# Blood pressure variability and early neurological outcomes in acute and subacute stroke in Southwestern Uganda

**DOI:** 10.1016/j.ensci.2023.100482

**Published:** 2023-10-16

**Authors:** Nicholas Kulaba, Adrian Kayanja, Denis Serubiri, Mark Kaddu Mukasa, Martin Kaddumukasa, Jane Nakibuuka, Shirley M. Moore, Elly T. Katabira, Martha Sajatovic, Cumara B. O'Carroll, Anthony Muyingo

**Affiliations:** a Department of Medicine, Mbarara University of Science and Technology, Uganda; b Department of Medicine, School of Medicine, College of Health Sciences, Makerere University, Uganda; c Neurological and Behavioral Outcome Center, University Hospitals Cleveland Medical Center, Case Western Reverse University School of Medicine, 11100 Euclid Avenue, OH 44106, USA; d Department of Neurology, Mayo Clinic College of Medicine and Science, 5777 East Mayo Blvd, Phoenix, AZ 85054, USA

**Keywords:** Blood pressure variability, Stroke, Outcomes, Cerebral infarction, Intracerebral hemorrhage, Sub-Saharan Africa

## Abstract

**Background:**

Greater blood pressure variability has detrimental effects on clinical outcome after a stroke; its effects are controversial and have not been evaluated in Sub-Saharan Africa (SSA).

**Methods:**

We conducted a prospective study of patients with CT head confirmed ischemic and hemorrhagic strokes admitted to a tertiary hospital within 7 days of onset of unilateral neurological deficits. Blood pressure variability indices, standard deviation (SD) and coefficient of variation (CV) of systolic and diastolic blood pressure between day 0 and day 7, were calculated with a subsequent modified Rankin Scale (mRS) score on day 14 post-stroke. Linear regression was performed to determine the exponential coefficients of mortality at 14 days post- stroke.

**Results:**

Out of 120 patients, 51.7% were female, 52.5% had ischemic stroke and the overall median age was 65 (IQR 54–80) years. Twenty (16.7%) patients died within a median survival time of 7 days, while 32 (26.7%) died by day 14 post-stroke. Patients with hemorrhagic stroke had an overall SDSBP of 16.44 mmHg while those with ischemic stroke had an overall SDSBP of 14.05 mmHg. In patients with ischemic stroke, SDSBP had adjusted coefficients of 1, *p* = 0.004 with C·I: 1.01–1.04 and NIHSS had adjusted coefficients of 1, *p* = 0.019 with C·I: 1.00–1.03 while in patients with hemorrhagic stroke, SDSBP had adjusted coefficients of 1, *p* = 0.045 with C·I: 1.00–1.04 and NIHSS had adjusted coefficients of 1, *p* ≤0.001 with C·I: 1.01–1.03.

**Conclusion:**

Exponential increase in Blood Pressure Variability (BPV) and stroke severity scale were independently associated with early mortality among all stroke patients in our study. We recommend future studies to evaluate whether controlling BPV among patients with stroke in Sub-Saharan Africa can reduce mortality.

## Highlights

• Patients with acute and subacute stroke in southwestern Uganda have high blood pressure variations.
• High blood pressure variations are associated with poor stroke clinical outcomes in Ugandan patients.
• Association of blood pressure variability with neurological outcomes after stroke is not linear.
• Reverse causality between blood pressure variability and outcome cannot be excluded.
• Severe stroke is associated with greater disturbance in the autonomic nervous system.

## List of abbreviations

**Table:** 

BP	Blood Pressure
BPV	Blood Pressure Variability
CT	Computed tomography
CV	Coefficient of Variation
CVA	Cerebrovascular accident
MRRH	Mbarara Regional Referral Hospital
mRS	modified Rankin scale
NIHSS	National Institutes of Health Stroke Scale
SD	Standard Deviation
SDDBPV	Standard Deviation Diastolic Blood Pressure Variability
SDSBPV	Standard Deviation Systolic Blood Pressure Variability

## 1 Introduction

Stroke results in both chronic disability and death [1]. The 2019 update on global stroke statistics highlighted a steady rise in stroke occurrence in low- and middle-income countries (LMICs) with Africa having up to 2–3-fold higher rates of stroke than Western Europe. In recent decades, studies have shown that Africa has an annual stroke incidence of 316 per 100,000 and a 3-year fatality rate >80% [2]. The case fatality among patients with stroke at 30 days ranges from 16.2% to 46% in hospital-based studies from Africa [2]. In Uganda, studies have shown a high 30-day mortality at 26.8% - 38.1% [[3], [4], [5]]. An increase in stroke admissions is linked to a growing population with high burden of uncontrolled hypertension, which is a key modifiable risk factor [6,7]. In Africa, most patients with stroke are found to have high blood pressure [8]. Increased blood pressure variability is associated with worse neurological outcomes and death among patients with acute stroke [[9], [10], [11]]. Blood pressure (BP) changes occurring immediately after ischemic stroke aim to restore impaired cerebral autoregulation and represents a response that maintains cerebral blood flow and perfusion to the ischemic penumbra [12,13]. These BP changes impair tissue oxygenation causing injury to the brain, while excessive perfusion results in the breakdown of the blood brain barrier [14].

The excessive rise in BP in patients with hemorrhagic stroke via the Cushing reflex and major stroke related stress [14,15] can lead to hematoma expansion which may result in neurological deterioration, while in ischemic stroke can potentially increase the risk of hemorrhagic transformation [16]. Both systolic and diastolic BPs usually rise immediately after stroke then fall in the following 7–10 days with the greatest drop in the first 1–2 days [13]. Greater blood pressure variability has cumulative detrimental effects on clinical outcome after a spontaneous intracerebral hemorrhage due to its effects on hematoma expansion, edema expansion and increased intracranial pressure [17].

The association of BPV with neurological outcomes after stroke is not linear and warrants further investigation. The magnitude to which systolic BP lowering influences outcome has been linked heavily on the size of the hematoma [18]. The reverse causality between BPV and outcome cannot be excluded as severe stroke has been associated with greater disturbance in the autonomic nervous system resulting in higher BP fluctuations [18]. In Sub-Saharan Africa, there are no studies that have evaluated the effect of blood pressure variability among patients with stroke and its effect on clinical outcomes. Due to this gap in practice, we designed a cohort study to determine the effect of BPV on early clinical outcome among patients with stroke presenting to a tertiary hospital in Southwestern (SW) Uganda.

## 2 Methods

### 2.1 Study design and participants

This study was a prospective cohort of patients with acute and subacute stroke admitted to Mbarara Regional Referral Hospital, a tertiary hospital in SW Uganda. We included patients 18 years of age and older with sudden onset of unilateral neurological deficits within 7 days, and non-contrast Computerized Tomography (CT) head confirmation of ischemic or hemorrhagic stroke. We excluded patients with traumatic intracerebral hemorrhage such as subdural hematomas, epidural hematomas, and traumatic brain injury and those who died or were lost to follow up before completion of one day of blood pressure measurement. On admission, all patients were positioned with the head of the bed elevated at 30 degrees to prevent aspiration, and oxygen saturation was kept above 93% as the standard of care. An initial blood pressure was measured using EDAN M3® (Edan USA 2014). Three blood pressure values were taken on admission and the average of the last two was considered the blood pressure at hospital admission. [19]. Socio-demographics (e.g. age, sex, marital status) and lifestyle factors (i.e. smoking and alcohol history) were captured. Past medical records were evaluated to capture history and duration of hypertension, diabetes mellitus, types of medications prescribed, presence of co-morbid kidney disease and heart disease. A complete clinical examination was conducted which included the Mayo Clinic Full Outline of Unresponsiveness (FOUR) score to evaluate the level of consciousness and the National Institutes of Health Stroke Scale (NIHSS) score to assess stroke severity. Stratification of the NIHSS score was as follows: 1–4 = minor stroke, 5–15 = moderate stroke, 16 to 20 = moderate to severe stroke, and 21–42 = severe stroke [20].

### 2.2 Laboratory procedures

Capillary blood glucose was measured using Accuchek glucometer (Roche Diagnostics Inc.). Total Cholesterol (TC) was measured using an enzymatic linked immunosorbent assay method in a Human 200 analyzer (German Design, Human Diagnostics), renal function tests, serum sodium and potassium were measured using Sysmex XNL-550®.

### 2.3 Blood pressure monitoring

After the initial clinical evaluation, admission blood pressure was measured, then follow-up blood pressures were measured at intervals of 6 h (6 am, noon, 6 pm and midnight) using a standard noninvasive automated BP monitoring device EDAN M3® (EdanUSA 2014) on the non-paralytic arm up to day 7 post-stroke. The measurement interval was adopted from the European Cooperative Acute Stroke Trial (ECASS) which investigated the characteristics of blood pressure profiles as predictors of long-term outcome after acute ischemic stroke [21]. Each time the blood pressure was measured, 2 readings were taken, and both mean systolic and diastolic blood pressure were calculated and subsequently recorded as the blood pressure of the patient.

### 2.4 Outcome measures

The primary outcome was defined as 14-day mortality post-stroke. Additionally, a modified Rankin Scale (mRS) score, which is a measure of the degree of neurological disability was assessed.

### 2.5 Ethical considerations

The study was approved by the Institutional Review Board (IRB) at Mbarara University of Science and Technology (ID: MUST-2021–118) and Uganda National Council of Science and Technology (ID: HS1973ES). Participants that had capacity to consent provided written informed consent and in those that did not have capacity, consent was obtained from a surrogate decision maker.

### 2.6 Statistical analysis

Clinical characteristics were computed as mean, and standard deviation for normally distributed variables. Categorical variables were summarized in frequencies and percentages. We used a student's *t*-test for continuous variables and chi-square test to demonstrate a difference in the baseline characteristics between patients with hemorrhagic stroke and ischemic stroke. Blood pressure variability was expressed as Standard Deviations (SD) (SDSBP), and Coefficient of variation (CV) (CVSBP) of Systolic and Diastolic BP respectively.

SD = √ ∑ (SBP- SBP _mean_) ^2^ /n and CV = [(SDSBP/ BP _mean_)] 100]. Linear regression analysis was fitted separately for each type of stroke, adjusted for baseline sociodemographic and clinical characteristics like age, NIHSS, admitting systolic blood pressure, random blood sugar, SDSBP, C-reactive protein and creatinine with the clinical outcome (14-day mortality) at 5% level of significance to determine the coefficients, 95% confidence interval and p - values. Exponential coefficients, *p*-values and their 95% confidence intervals were captured

## 3 Results

**Fig. 1:**
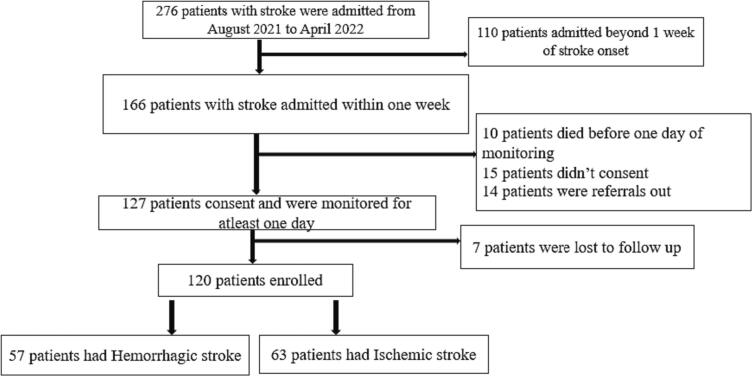
Study flow chart.

**Fig. 2:**
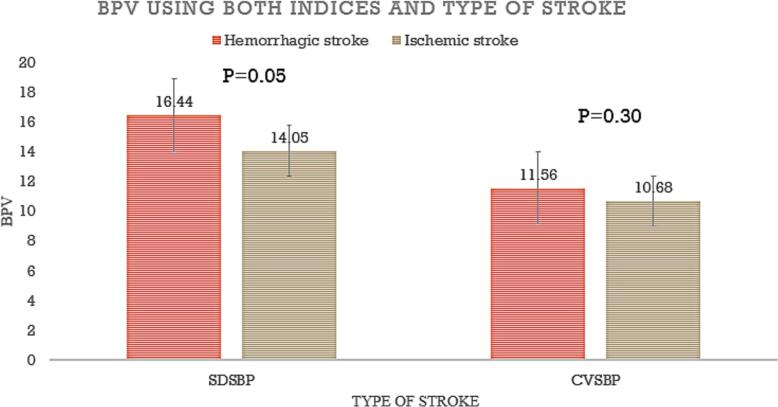
Blood Pressure Variability using both indices and type of stroke. BPV- Blood Pressure Variability, CVSBP- Coefficient of Variation Systolic Blood Pressure, SDSBP- Standard Deviation Systolic Blood Pressure.

We screened 276 eligible patients with unilateral neurological deficits between August 2021 and April 2022. We enrolled 120 patients with confirmed strokes on CT head (Fig. 1); 52.5% had ischemic stroke and 47.5% had hemorrhagic stroke (Table 1). Out of 120 patients, 51.7% were female, 10.8% had diabetes mellitus, 43.3% had hypertension with 21.7% using anti-hypertensive medication, and 7.5% were HIV positive. The overall median age was 65 years (IQR: 54–80). A history of smoking and excessive use of alcohol was elicited in 23.3% and 40.8% of all patients respectively. Patients with ischemic stroke were older, had diabetes mellitus and a high blood sugar on admission, compared to patients with hemorrhagic stroke who had higher NIHSS scores, higher mean systolic blood pressure and higher mean diastolic blood pressure on admission (Table 1). Patients with hemorrhagic stroke had statistically significant higher mean SDSBP (16.44 mmHg) compared to ischemic stroke (14.05 mmHg) with a *p* = 0.05. There was no statistical difference between hemorrhagic and ischemic stroke with mean SDDBP (9.89 mmHg vs 9.06 mmHg) with *p* = 0.19 respectively. There was no difference in CVSBP (11.56 mmHg vs 10.68 mmHg with a *p* = 0.30) and CVDBP (11.89 mmHg vs 11.64 mmHg with *p* = 0.75) between hemorrhagic and ischemic stroke (Fig. 2).

**Table 1:** Baseline clinical characteristics of patients with stroke.

Variable	Overall (*N* = 120)	Hemorrhagic stroke (*N* = 57)	Ischemic stroke (*N* = 63)	p-value
Sex Female n (%)	62 (51.7%)	26 (45.6%)	36 (57.1%)	0.21
Age (Mean ± SD)	66 ± 16	63 ± 15	68 ± 16	0.06
Married n (%)	85 (71%)	44 (77%)	41(65%)	0.15
Time to presentation in days (Median, IQR)	4 (3–5)	3 (3–5)	4 (3–5)	0.15
Diabetes Mellitus n (%)	13 (11%)	3 (5%)	10 (16%)	0.06
Hypertension n (%)	52 (43%)	29 (50.9%)	23 (36.5%)	0.11
Smoking n (%)	28 (23%)	12 (43%)	16 (57%)	0.57
HIV n (%)	9 (8%)	3 (33%)	6 (67%)	0.37
Alcohol n (%)	49 (41%)	26 (53%)	23 (47%)	0.31
NIHSS (median, IQR)	17.5 (11–24)	19 (12–26)	15 (9–22)	**0.02**
Four score (mean ± SD)	14 ± 2	13 ± 3	14 ± 2	**0.02**
SBP (mmHg) (mean ± SD)	153 ± 28	159 ± 27	147 ± 28	**0.02**
DBP (mmHg) (mean ± SD)	90 ± 18	94 ± 17	87 ± 19	**0.03**
RBS (mmol/L) (mean ± SD)	7.9 ± 3	7.7 ± 3.5	8.1 ± 3.3	0.59
CRP (mg/L) (mean ± SD)	67 ± 65	72.7 ± 67.6	61.8 ± 62.4	0.37
Neutrophil (mean ± SD)	6.8 ± 4	7.6 ± 4.3	6.0 ± 3.4)	**0.03**
T-chol (mg/dl) (mean ± SD)	164.7 ± 82.7	168.9 ± 87	161.2 ± 79	0.62
Crea (mg/dl) (Mean ± SD)	1.5 ± 1.1	1.73 ± 1.5	1.20 ± 0.4	**0.01**
NLR (Mean ± SD)	5.3 ± 5.4	6.44 ± 6	4.21 ± 4	**0.03**
Atrial fibrillation	3 (3%)	1 (1.8%)	2 (3.2%)	0.62
Antihypertensives	63 (53%)	43 (75.4%)	20 (31.7%)	**<0.001**

Crea - Creatinine, CRP- C-Reactive Protein, DBP- Diastolic Blood Pressure, HIV- Human Immunodeficiency Virus, NIHSS-National Institute of Health Stroke Scale, NLR- Neutrophil-Lymphocyte Ratio, RBS- Random Blood Sugar, SBP- Systolic Blood Pressure, SD- Standard Deviation, T-chol- Total cholesterol.

### 3.1 Primary outcome

The overall 14-day mortality for both hemorrhagic and ischemic stroke patients was 26.7% (32/120). The 14-day mortality was higher in hemorrhagic stroke (33.3%) than in ischemic stroke (20.6%).

Linear regression model was fitted to determine the association of blood pressure variability with 14 day mortality among patients with hemorrhagic and ischemic stroke separately. We found that every 1 mmHg exponential increase in the SDSBPV resulted in increased likelihood of mortality and additionally every 1 unit increase in stroke severity scale resulted in an increase in mortality for both ischemic stroke and hemorrhagic stroke. In patients with hemorrhagic stroke, stroke severity (NIHSS) had a coefficient of 1, *p*-value <0.001 with confidence interval (C·I) of (1.01–1.04) and SDSBP had a coefficient of 1, p-value 0.045 (C·I: 1.00–1.04). In ischemic stroke patients, stroke severity (NIHSS) had a coefficient of 1, p-value 0.019 (C·I: 1.00–1.03) (Table 2) and SDSBP had a coefficient of 1, p –value 0.004 (C·I: 1.10–1.04) (Table 3).

**Table 2:** Factors associated with 14 day mortality among patients with hemorrhagic stroke.

Variable	Bivariate (un adjusted)			Multivariate (adjusted)		
	Coe	95%C·I	P-value	Coe	95%CI	P-value
Age	1.0	0.99–1.01	0.175	0.9	0.99–1.01	0.985
Admitting Systolic BP	1.0	0.99–1.01	0.302	1.0	0.99–1.01	0.724
NIHSS	1.0	1.02–1.04	<0.001	1.0	1.01–1.04	**<0.001**
Baseline RBS	1.0	0.97–1.06	0.454	1.0	0.98–1.05	0.787
SDSBP	1.0	1.02–1.05	<0.001	1.0	1.00–1.04	**0.045**
Baseline CRP	1.0	0.99–1.00	0.275	0.9	0.99–1.00	0.205
Baseline Creatinine	1.1	1.00–1.17	0.050	1.0	0.98–1.09	0.142

**Table 3:** Factors associated with 14 day mortality among patients with Ischemic Stroke.

Variable	Bivariate (un adjusted)			Multivariate (adjusted)		
	Coe	95%C·I	P-value	Coe	95%CI	P-value
Age	0.9	0.99–1.01	0.869	0.9	0.98–1.00	0.114
Admitting Systolic BP	1.0	0.99–1.01	0.723	0.9	0.99–1.00	0.960
NIHSS	1.0	1.01–1.03	<0.001	**1.0**	**1.00–1.03**	**0.019**
Baseline RBS	0.9	0.97–1.03	0.944	1.0	0.97–1.03	0.823
SDSBP	1.0	1.01–1.05	0.001	**1.0**	**1.01–1.04**	**0.004**
Baseline CRP	1.0	1.00–1.01	0.027	1.0	1.99–1.00	0.316
Baseline Creatinine	0.9	0.71–1.24	0.658	0.9	0.76–1.12	0.420

BP- Blood pressure, NIHSS-National Institute of Health Stroke Scale, RBS- Random Blood Sugar, SDSBP- Standard deviation Systolic Blood Pressure.

## 4 Discussion

In this study our objective was to determine the magnitude of blood pressure variability (BPV) measured using standard deviation (SD) and coefficient of variation (CV) among patients with acute and subacute stroke, and to distinguish any differences in the various measures of BPV on early neurological outcome measured by mRS at day 14 post-stroke. We also wanted to determine other factors associated with 14 days mortality among acute and subacute stroke. We found that increasing SDBP and NIHSS did significantly impact outcome, with greater blood pressure variability and stroke severity resulting in higher mortality. There was higher overall BPV and stroke severity (determined by NIHSS) among patients with hemorrhagic stroke compared to those with ischemic stroke. However, in both types of stroke, every 1 mmHg exponential increase in SDSBP resulted in increased mortality. We also found that every 1point increase in the NIHSS score resulted in increased mortality in both types of stroke. These findings confirm earlier reports of greater risk of death and/or dependence after acute stroke with rising SDBPV and NIHSS. Blood pressure variability indices (SD and CV) have been assessed in many post-stroke studies and are good estimates of BPV [18]. To the best of our knowledge, this is the only study in sub-Saharan Africa that has evaluated BPV in stroke, correlating it with 14-day post-stroke mortality.

In our cohort, patients with hemorrhagic stroke had higher overall BPV in both systolic and diastolic blood pressure compared to ischemic stroke. However, our BPV values are much lower compared to those of Fischer et al. where a SDSBP of 37.6 mmHg in hemorrhagic stroke and a 30.5 mmHg in ischemic stroke was measured in the first 24 h of stroke onset. Patients with hemorrhagic stroke are more likely to have aggressive in-hospital interventions to lower BP compared to ischemic stroke patients due to the biological rationale to reduce or even tamponade the ongoing bleeding and reduce the size of the haematoma in the brain [22,23]. These interventions to lower blood pressure in patiemts with hemorrhagic stroke may be responsible for the higher BPV (greater deviation from the initial mean BPs) than those seen for patients with ischemic stroke. Using different methods of blood pressure assessment likely contribute to disparate results. For example, the use of an automated BP monitor in our study at fixed intervals versus ambulatory BP monitoring and invasive BP monitoring in other studies, and the fact that BP measurements were taken within 24 h of stroke in the previous studies compared with up to 7 days in our study, may explain some of the differences. The automated BP monitor likely underestimates the mean arterial pressure (MAP) and diastolic blood pressure compared with intra-arterial measurements, but SBP values may be similar [24].

In our cohort of patients, we found an overall mortality of 26.7%, which is high indicating that up to a quarter of the patients in our cohort died. The mortality in our limited-resource setting is much higher compared to other studies conducted in high-income countries [25,26]. Hemorrhagic stroke contributed the highest mortality in our cohort. There is evidence that raised BP levels in acute hemorrhagic stroke is associated with a worse prognosis [27]. In hemorrhagic stroke, high blood variability increases hematoma expansion thus worsening the clinical outcome of patients [28] while in ischemic stroke, the penumbra is particularly sensitive to negative effects of cerebral perfusion fluctuations caused by high blood pressure variability [29]. Rapid falls in blood pressure increase the peril of tissue ischemia expansion and reduce the chance of reperfusion, while a rapid increase in blood pressure increases the risk of hemorrhagic transformation [30]. Notably, hemorrhagic stroke patients in our study also had statistically significant higher NIHSS, coma FOUR scores, and serum creatinine at baseline, all of which may be associated with a higher mortality in this population. Well designed and larger studies may be needed to further explore this relationship to guide care in our settings.

In our study we found that a 1 point increase in the NIHSS score on admission resulted in increased likelihood of mortality among patients with both hemorrhagic and ischemic stroke. Our findings are consistent with previous studies confirming that severe stroke at admission predicts mortality [4,5]. In similar studies conducted in Uganda, stroke patients presented in a delayed time period and with higher than expected NIHSS scores. Delays in presentation are likely multifactorial in the setting of limited community resources, lower levels of education, less awareness of stroke symptoms, long distances from a referral hospital or higher-level health center, and transportation challenges. The delayed presentation may be contributing to the higher stroke severity on admission, but we must also contemplate a selection bias considering that patients with minor stroke are probably not presenting to hospitals in order to be evaluated. Furthermore, patients with severe stroke are at a high risk of complications like aspiration pneumonia, catheter related infections and dehydration hence increasing the likelihood of mortality [31].

Our study adds to the growing body of evidence demonstrating that greater BPV is related to negative cerebrovascular outcomes [10,32,33]. These findings are particularly important when considering options for BP reduction after stroke and which medications are more efficacious in reducing BPV. It is clear from these findings that single blood pressure measurements have limited prognostic value with regard to outcomes when compared to multiple measurements of BP [34]. Our analysis of BPV is based on repeated BP measurements at fixed intervals, and BPV was a strong predictor of higher mortality in stroke patients in SW Uganda. Larger-scale trials are needed to confirm our findings and explore the mortality and disability implications of successful and widespread interventions aimed at reducing BPV.

This study also had some limitations. Due to delayed presentation of patients to the referral hospital, this might have underestimated blood pressure variability measures considering that the highest blood pressure fluctuations typically occur within the first 72 h after stroke and we measured blood pressure variability up to 7 days post-stroke. This study only included patients with unilateral motor deficits which may limit generalizability of findings to patients with other deficits, such as isolated aphasia, sensory loss, visual field deficits and ataxia. We also excluded patients who died before 24 h of blood pressure monitoring thus potentially excluding patients with more severe strokes on presentation. Patients with small ischemic strokes were less likely represented in our study since there had to be CT head confirmation of stroke before inclusion, therefore potentially excluding patients with clinical findings of ischemic stroke but not reaching the threshold for CT detection. There is a concern that baseline clinical features were worse among patients with hemorrhagic strokes and the related impact on the mortality rates could not be explicitly explained. The impact of the clinical features on mortality cannot be determined with precision from the available data, further well designed studies may be needed.

In conclusion, we have provided evidence that high BPV measured in both ischemic and hemorrhagic stroke is associated with poor stroke outcome in SW Uganda. We hope that these findings serve as a catalyst for further studies evaluating whether or not controlling BPV among patients with stroke can reduce mortality and disability.

## Disclosures

None.

## Funding

Research reported in this publication was supported by a grant from the National Institutes Health (1R01NS118544-01): 10.13039/100000065National Institute of Neurological Disorders and Stroke (NINDS) 10.13039/100000061Fogarty International Center (FIC) to Martha Sajatovic and Elly T. Katabira. The content is solely the responsibility of the authors, and does not necessarily represent the official views of the National Institute of Health.

## Authors' contributions

The authors named in this manuscript contributed substantially to this research work and met the criteria for authorship. Nicholas Kulaba, Adrian Kayanja, Cumara B. O'Carroll and Anthony Muyingo took part in the conception of the research idea, development of the research project, elaboration of the research protocols, and correction of the manuscript and approved the final manuscript. Mark Kaddu Mukasa, Martin Kaddumukasa, Jane Nakibuuka, and Shirley M. Moore took part in the correction of the research project and protocols, manuscript writing, and approval of the final manuscript. Denis Serubiri took part in data collection, data interpretation, manuscript revision, and approval of the final manuscript.

## CRediT authorship contribution statement

**Nicholas Kulaba:** Conceptualization, Data curation, Formal analysis, Investigation, Methodology, Writing – original draft, Writing – review & editing. **Adrian Kayanja:** Conceptualization, Formal analysis, Methodology, Supervision. **Denis Serubiri:** Data curation, Methodology. **Mark Kaddu Mukasa:** Funding acquisition, Methodology, Writing – original draft, Writing – review & editing. **Martin Kaddumukasa:** Funding acquisition, Methodology. **Jane Nakibuuka:** Funding acquisition, Methodology. **Shirley M. Moore:** Formal analysis, Funding acquisition, Writing – original draft, Writing – review & editing. **Elly T. Katabira:** Funding acquisition, Methodology. **Martha Sajatovic:** Funding acquisition, Methodology, Supervision. **Cumara B. O'Carroll:** Conceptualization, Formal analysis, Investigation, Methodology, Writing – original draft, Writing – review & editing. **Anthony Muyingo:** Conceptualization, Data curation, Formal analysis, Methodology, Supervision, Visualization, Writing – original draft, Writing – review & editing.

## Declaration of Competing Interest

The authors declare that they have no competing interests.
